# High-throughput sequencing and degradome analysis reveal neutral evolution of *Cercis gigantea* microRNAs and their targets

**DOI:** 10.1007/s00425-015-2389-y

**Published:** 2015-09-05

**Authors:** Wenna Guo, Ying Zhang, Qiang Wang, Yueping Zhan, Guanghui Zhu, Qi Yu, Liucun Zhu

**Affiliations:** School of Life Sciences, Shanghai University, Shanghai, 200444 People’s Republic of China; Yangzhou Breeding Biological Agriculture Technology Co. Ltd, Yangzhou, 225200 People’s Republic of China; State Key Laboratory of Pharmaceutical Biotechnology, School of Life Sciences, Nanjing University, Nanjing, 210093 People’s Republic of China; Department of Radiation Oncology, Fudan University Shanghai Cancer Center, Shanghai, People’s Republic of China; Department of Oncology, Shanghai Medical College, Fudan University, Shanghai, People’s Republic of China; Department of Radiation Oncology, Shanghai Proton and Heavy Ion Center, Fudan University Cancer Hospital, Shanghai, China

**Keywords:** Degradome analysis, High-throughput sequencing, Legume, miRNA, Neutral evolution

## Abstract

**High-throughput sequencing and degradome analysis for*Cercis gigantea*identified 194 known miRNAs and 23 novel miRNAs with 61 targets. The comparison results of highly conserved miRNAs and non-conserved miRNAs implied that*C. gigantea*miRNAs were subjected to the neutral evolution**.

MicroRNAs play a key role in post-transcriptionally regulating gene expression during plant growth, development and other various biological processes. Although numerous miRNAs have been identified and documented, to date, there are no reports on *Cercis gigantea* (*C. gigantea*) miRNAs. In this study, *C. gigantea* miRNAs and their target genes were investigated by extracting RNA from young roots, tender stems, young leaves, and flower buds of *C. gigantea* to establish a small RNA and a degradome library to further sequence. This study identified 194 known miRNAs belonging to 52 miRNA families and 23 novel miRNAs. Among these, 158 miRNAs from 27 miRNA families were highly conserved and existed in a plurality of plants. In addition, 60 different targets for 30 known families and one target for novel miRNA were identified by high-throughput sequencing and degradome analysis in *C. gigantea*. The comparison results revealed that highly conserved miRNAs have higher expression levels, more family members and more targets than non-conserved miRNAs, indicating that *C. gigantea* miRNAs were subjected to the neutral evolution. Meanwhile, these conserved miRNAs were also found to be involved in auxin signal transduction, regulation of transcription, and other developmental processes, which will help further understanding regulatory mechanisms of *C. gigantea* miRNAs.

**Electronic supplementary material:**

The online version of this article (doi:10.1007/s00425-015-2389-y) contains supplementary material, which is available to authorized users.

## Introduction

*Cercis gigantea* is a tree belonging to the subfamily Caesalpinioideae of the Leguminosae. This species is endemic to China, and has a wide range of growth habitat, strong adaptability, resistance against pathogens and pests, a rapid growth rate, and a long lifespan. Moreover, *Cercis* is the first branch of the Leguminosae in the taxonomy tree, allowing it to act as a bridge that connects the legumes to other plant species. Therefore, understanding the mechanism of growth and development of *C. gigantea* may provide important information for other studies on legumes.

Investigation of gene expression and its regulatory mechanism is crucial for research in plant growth and development. Meanwhile, small RNA-guided regulation plays an important role in metabolism (Nag and Jack 2010), epigenetic control of transposable elements (Lisch 2013), hormone responses (Liu and Chen 2009), and responses to variety of stresses of plants (Liu et al. 2014). Among them, microRNAs play significant roles in post-transcriptional and translational gene regulation (Bartel 2009; Li et al. 2014). miRNAs are one of the most abundant small RNAs (sRNAs) in plants and animals, with typical lengths of 18–25 nucleotides. They are a group of endogenous non-coding sRNAs that regulate gene expression mainly via repressing the translation or mediating the cleavage of target mRNA at the post-transcriptional level (Moran et al. 2014). miRNAs were first discovered in 1993 (Lee et al. 1993). To date, 30,424 mature miRNA sequences from 206 species have been identified and included in the Sanger miRNA data base (miRBase, version 20.0). Numerous studies have shown that miRNAs are involved in various processes such as metabolism, growth and development of plants as well as biotic and abiotic stress tolerance where some miRNAs were induced to express in response to pathogen exposure, salt damage, drought, and nutrition deprivation (Ding et al. 2009; Yang et al. 2014). Examples include the following: miRNAs were reported to participate in regulating maize ears development (Liu et al. 2014); overexpression of miR160a occurred in *Oryza sativa* in response to *Pyricularia oryzae* infection (Li et al. 2014); different patterns of miRNA expression were observed in roots and stems of *Oryza sativa* due to phosphorus deficiency and recovery (Secco et al. 2013), and 19 miRNAs were down-regulated and 2 miRNAs were up-regulated in *Populus tremula* due to salt damage (Ren et al. 2013). However, miRNAs remain unknown in *C. gigantea* till now. Therefore, it is necessary to investigate *C. gigantea* miRNAs and their targets.

There are plentiful methods to detect miRNA targets, including computational predictions (Cheng and Li 2008), Argonaute (AGO) protein immunoprecipitation (Beitzinger et al. 2007), RNA ligase-mediated 5′rapid amplification of cDNA ends assay (RLM 5′RACE) (Hsieh et al. 2009), and miRNA microarray analysis (Lim et al. 2005) and luciferase assay (Liu et al. 2007). However, these methods have certain limitations, such as the very high false positive and false negative predictions in the computational method and the complex procedures required for the experimental methods, which are time-consuming and unable to accurately validate a mass of miRNA targets at the same time. With the development of high-throughput sequencing technology, a new detection method has emerged for miRNA targets, known as degradome sequencing technology, which combines the advantages of high-throughput deep sequencing, bioinformatics analysis, and RACE. In this technology, deep sequencing analyses are performed on target mRNA degradation fragments cleaved by miRNA to identify the miRNA targets (German et al. 2009). At present, this method has been successfully applied to study the miRNA targets in *Arabidopsis thaliana* (Addo-Quaye et al. 2008), rice (Sun et al. 2015) and other plants (Liu et al. 2014; Zhang et al. 2014).

In this study, we extracted RNA samples from young roots, tender shoots, young leaves, and flower buds of *C. gigantea* to perform sRNA and degradome sequencing, resulting in the 194 known miRNAs and 23 novel miRNAs, as well as 61 miRNA targets of *C. gigantea*. Compared to the other plant miRNAs, *C. gigantea* miRNAs could be classified as conserved and lineage-specific miRNAs, in which the conserved miRNAs family had more members and more miRNA targets, and their targets were also conserved across species. In addition, Gene Ontology (GO) analysis revealed involvement of *C. gigantea* miRNAs in the auxin signal transduction, regulation of transcription and other growing and developmental processes, which will help further investigating biological functions and regulatory mechanisms of *C. gigantea* miRNAs.

## Materials and methods

### Plant materials and RNA extraction

The samples were collected from the young roots, tender shoots, young leaves and flower buds of wild *C. gigantea* growing in Jiangsu Province. TRIzol reagent (Invitrogen) was used to extract the total RNAs (Hafner et al. 2008). Agilent 2100 Bioanalyzer Nanochips and NanoDrop 2000 Spectrophotometer (Agilent Technologies, Santa Clara, CA, USA) were then employed to evaluate the quality and quantity of the total RNAs (Kogenaru et al. 2012). The extracted total RNAs from the four tissues were mixed in equal and used in subsequent sequencing.

### High-throughput sequencing

Total RNAs were processed for construction and sequencing of the sRNA and degradome libraries as previously described (Liu et al. 2014; Zhang et al. 2014). An Illumina next-generation sequencing system, i.e., the 1 G Genome Analyzer sequencing platform, was utilized for sRNA sequencing. An Illumina HiSeq 2000 (LC Sciences, Houston, TX, USA) was used for degradome sequencing. Sequencing data are available in Gene Expression Omnibus (GEO) under the series accession GSE66754. This accession includes the results of sRNA and degradome sequencing of *C. gigantea*.

### Small RNA sequencing and identification of known and novel miRNAs

The entire process of Illumina sRNA data analysis is shown in Fig. S1. Redundant sequences, 3′ adapter sequences, sequences with lengths shorter than 17 nt and longer than 27 nt sRNA, and sRNA sequences that included junk reads were removed from raw data to obtain unique clean reads for identification of *C. gigantea* miRNAs. Redundant sequences here refer to 100 % sequence identity compared to other sequences. We first calculated the counts of redundant sequences, then ordered the sequences by the counts, and finally saved the name of sequences with their order and copy number. And the “junk reads” were defined as the reads that included more than one unknown bases, or seven bases A, or seven bases T, or eight bases C, or six bases G, or reads including more than nine dimer, or five trimer, or four tetramer.

The miRBase includes 6843 miRNAs from 72 plant species. To identify the known miRNAs (miRNAs which have been identified in other species) from *C. gigantea*, unique clean reads were used to query the miRBase (−r 5, −W 4, E-value <1) that fulfilled any of the following criteria: (a) perfect match with the miRNA sequence or its reverse complementary sequence; (b) exact match with the miRNA seed sequence, with an identity ≥95 %, length matching ≥90 % of the read, and read abundance ≥5; (c) not exactly matching the sequence, with a similarity ≥95 %, length matching ≥90 % of the read, and read abundance ≥10.

To find the novel *C. gigantea* miRNAs, the previous transcriptome sequencing data were re-assembled and the length of 70–200-bp transcripts was used as the candidates of the pre-miRNAs. To make sure the novel miRNA would be more authentic, we applied rigorous criteria to these sequences to eliminate spurious miRNAs as much as possible. The unique clean reads with high abundance (>5) were first located on the transcripts using BLASTN program (E-value <1). The RNA secondary structure prediction software (RNAFold) was then employed to determine whether the transcripts with an exact sRNA match had a stem-loop structure (Dutta et al. 2014). Their minimum free energy (MFE) and adjust minimal folding free energy (AMFE) were also measured. The novel miRNA candidate was determined when the stem-loop structure with miRNA candidate located in the arm was required, adjust minimum free energy was less than −15 kcal/mol, and mismatches between miRNA and the complementary strands of functional mature miRNAs, miRNA* were no more than 4. Finally, Rfam online database (Burge et al. 2013) was used to remove other types of sRNAs (rRNAs, scRNAs, snoRNAs, snRNAs, and tRNAs) to obtain the pre-miRNA sequence and the novel miRNA of *C. gigantea*. In addition, miRDeep-P (Yang and Li 2011; Jain et al. 2014) was applied on our data as well to access the accuracy of the above method we used to identify novel miRNAs.

### Identification of miRNA targets

The identified *C. gigantea* miRNAs were mapped to the transcript using BLASTN. Then, each miRNA and mRNA match was scored according to the following rules: Mismatch: 1 score deduction; G:U match: 0.5 score deduction; and if the above two conditions happened on the 2nd–13th positions at 5′ end of the miRNA: double the deduction score. The mRNA was identified as a candidate miRNA target if the abundance of degradome reads at the mRNA cleavage site was no less than 5; the miRNA and its target mRNA sequences were reverse complementary, and the total of the deduction scores was less than 4 (Wang et al. 2011). Meanwhile, the CleaveLand pipeline (Addo-Quaye et al. 2009) was also used to identify miRNA targets. The consistent mRNAs obtained from both methods were chosen as miRNA targets.

### Annotation

The soybean and *Arabidopsis thaliana* protein sequences as references were downloaded from the UniProt and Ensembl plant database. The sequences of *C. gigantea* miRNA targets were adopted as queries in local BLASTX searches for potential orthologs in the soybean and *Arabidopsis thaliana* protein database (E-value <1.0e-5). The functional annotation of *C. gigantea* miRNA targets was determined using DAVID online software (Huang da et al. 2009). The GO annotations of the targets were obtained from our previous study.

### Analysis of evolutionary pattern of miRNA targets

In this study, miRNA expression level represents as the copy number of corresponding unique clean reads. Nucleotide divergence between orthologs was evaluated by nucleotide divergence (Pi) with the Jukes and Cantor correction (Chen et al. 2010). Ks and Ka, which were counted based on Nei and Gojobori (Zhu et al. 2013), representing the number of synonymous substitution per synonymous site and the number of nonsynonymous substitutions per nonsynonymous site, respectively. Generally speaking, the ratio of Ka/Ks greater than 1 implied positive selection, and the ratio less than 1 suggested negative selection (Chen et al. 2010).

### Definition of conserved and non-conserved miRNAs

In this study, the miRNA families fell into four classes based on their level of conservation. In Class I, the miRNA families were present in both dicotyledons and monocotyledons, defined as the highly conserved miRNA family. If the miRNA families were just identified in dicotyledons, they were defined as the relatively conserved miRNA family belonging to Class II. It was grouped as Class III when the miRNA families were only present in legumes, which we named as the relatively non-conserved miRNA family. And in Class IV, the miRNA families only appeared in *C. gigantea*, defined as the species-specific miRNA family of *C. gigantea*. Among the four classes, miRNAs from Class I, II, III were known miRNAs, while miRNAs from Class IV were novel miRNA, miRNAs from Class I and Class IV were also known as highly conserved miRNAs and species-specific miRNAs, respectively; miRNAs from both of Class III and IV were called genus-specific miRNAs. Meanwhile, miRNAs from Class II, III and IV were defined as non-conserved miRNAs in this study.

## Results

### Overview of small RNA library sequencing

**Fig. 1:**
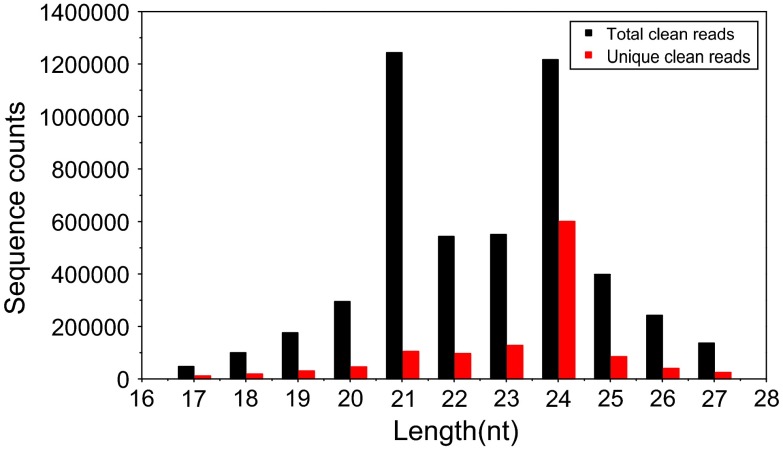
Length distribution and abundance of sRNA reads

To identify miRNAs in *C. gigantea*, the total RNA samples were extracted from young roots, tender shoots, young leaves and flower buds of *C. gigantea* for sRNA sequencing to return 5,270,698 raw reads. After data processing, a total of 1,191,483 unique clean reads were obtained with lengths ranging from 17 to 27 nt (Table 1). Figure 1 shows the length distribution of redundant clean reads and unique clean reads. The majority of the redundant sRNAs (71.2 %) were 21–24 nt in length, which is consistent with the typical size distribution of dicer-derived products and previous studies on sRNAs of soybean (Xu et al. 2013), *Arabidopsis thaliana* (Lu et al. 2008b) and grapevine (Pantaleo et al. 2010).

**Table 1:** Summary of data from *Cercis gigantea* small RNA sequencing

Type	Number of reads	Percentage (%)
Total unique reads	1,349,747	100
17–27 nt	1,191,483	88.27
Junk reads: ≥2N, ≥7A, ≥8C, ≥6G, ≥7T	7,202	0.534
Junk reads : ≥10Dimer, ≥6Trimer, ≥5Tetramer	1,022	0.076
Unique clean reads	1,183,260	87.67

### Identification of known miRNAs

**Fig. 2:**
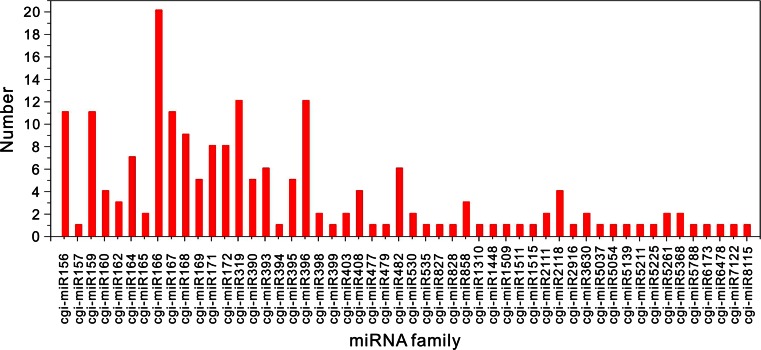
Number of miRNAs for each miRNA family in *Cercis gigantea*

**Fig. 3:**
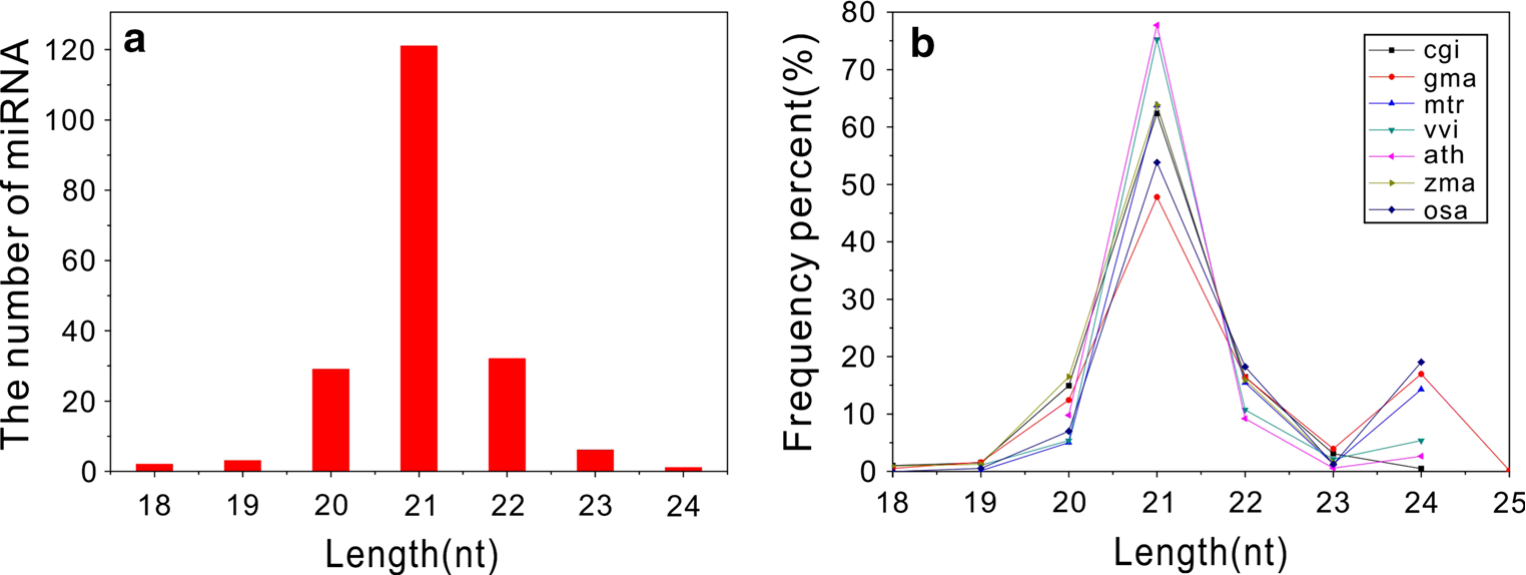
Length distribution of miRNAs. **a** Length distribution of known miRNAs in *Cercis gigantea*. **b** Length distribution of the miRNAs in *Cercis gigantea* and six plants and in miRBase database (*Glycine max*, *Medicago sativa*, *Vitis vinifera*, *Arabidopsis thaliana*, *Zea mays*, *Oryza sativa*)

**Fig. 4:**
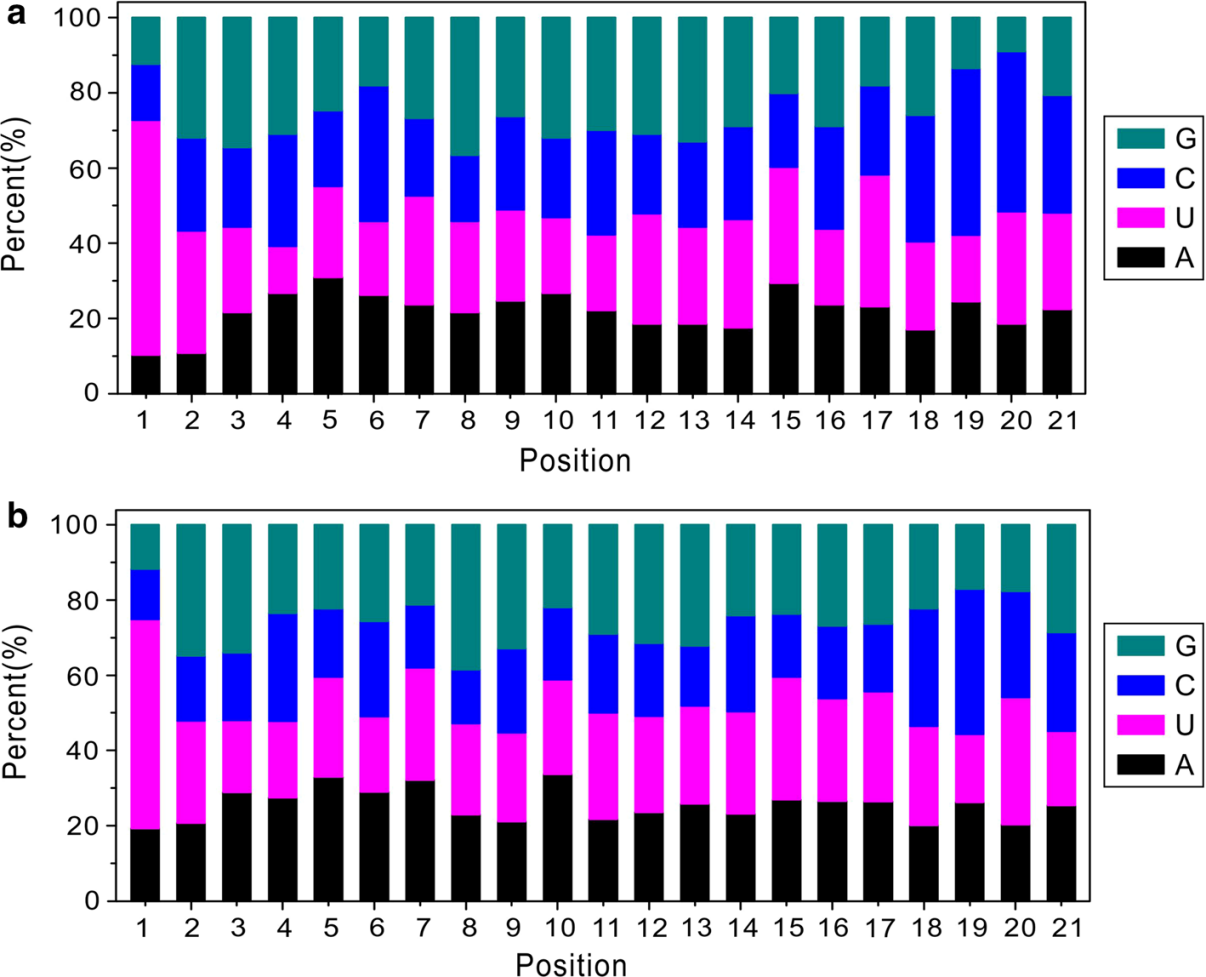
Nucleotide bias at each position of miRNAs. **a** The nucleotide bias of known miRNAs in *Cercis gigantea*. **b** The nucleotide bias of the total plant miRNAs in miRBase database

Compared to the known miRNA of all plant species in miRBase, we identified a total of 1014 unique clean reads corresponding to 194 known miRNAs from 52 miRNA families. The number of miRNA family members varied greatly, as shown in Fig. 2. The miRNA expressions were significantly different among various miRNA families as well, with changes in the copy number of corresponding reads ranging from 2 (miR828) to 642,182 (miR166). Similarly, the expression of different miRNA family members within the same miRNA family also varied largely. For example, the copy number of corresponding reads of various members in the miR166 family was tremendously different from each other, which ranged from 1 to 59,024 (Table S1). Furthermore, the length distribution of miRNAs showed that the 21-nt miRNA was the most abundant, accounting for 61.9 %, in line with the length distribution of plant miRNAs in miRBase (Fig. 3). To understand whether the base preference of *C. gigantea* miRNAs existed, the base distribution for each position in known miRNAs was performed. The result revealed that U mostly appeared at the 5′ end of the miRNA (62.4 %, seen in Fig. 4), which agreed with the base preference of miRNAs in other plants.

### Identification of species-specific miRNAs

One of the advantages for high-throughput sequencing is to discover novel miRNAs. A total of 23 novel miRNAs were detected with pre-miRNA lengths ranging from 72 to 186 bps and adjust minimum free energy ranging from -15.98 to -56.02 kcal/mol (Table S2). The length distribution of the novel miRNAs showed that the 24-nt miRNA was the most abundant (39.1 %, Fig. S2), and U appeared mainly at the 5′ end of novel miRNAs as well (52.7 %, Fig. S3). These results are consistent with known miRNAs in *C. gigantea* (Figs. 3a, 4b). To make sure these novel miRNAs are of high confidence, we also detected the star sequence of the mature miRNA in our data. The star sequences that expressed at lower level than their corresponding miRNAs were observed, which is in accordance with the report that star sequences are degraded and usually occur at significantly lower levels (Creighton et al. 2010).

Furthermore, four novel miRNAs were identified by miRDeep-P, three of them were consistent with our results, and the other one which had five nucleotide differences between mature and star miRNA was not included in our result (larger than the criterion in out methods). Although three of these four miRNAs were also found in our study, the number of the miRNAs predicted by miRDeep-P was much lower than expected since Jain et al. (2014) had identified 120 novel miRNAs in chickpea using this software with genomic sequences as the reference. To determine the reason for different number of identified miRNAs between the two approaches, the sRNA sequencing data (GSE51300) and the transcriptome sequencing data (SRR627765) of chickpea (Jain et al. 2014) were downloaded from GEO database and SRA database, respectively. The transcriptome data were assembled into transcripts by Trinity. MiRDeep with same criteria in the previous study (Jain et al. 2014) was used to identify chickpea miRNAs on transcriptome data. The results revealed that only 8 novel candidate miRNAs were identified when setting transcriptomic sequences as reference, which was also much less than 120 novel candidate miRNAs that identified using genomic sequences as reference. It is clear that more candidate miRNAs could be found with our method when genomic sequence was unavailable.

### Target prediction of miRNAs using degradome sequencing

**Fig. 5:**
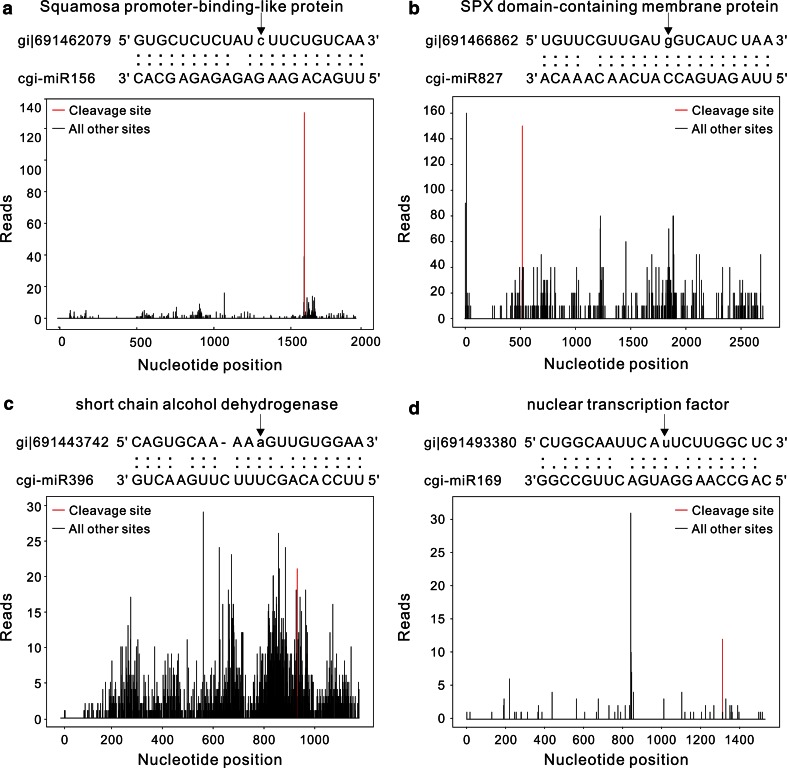
Target plots (*t* plots) of identified miRNA targets in the three different categories using degradome sequencing. The T plots show the distribution of the degradome tags along the full length of the target mRNA sequence (*bottom*). The red line represents the miRNA cleavage of target transcripts. The alignments show the miRNA with a portion of its target sequence (*top*). *Two dots* matched RNA base pairs; *one dot* a GU mismatch. The lower case nucleotide on the target transcript represents the cleavage site, shown by an *arrow*. **a** Example of cgi-miR156 slicing target gi|691462079 at nt 1602 for category 0. **b** Example of cgi-miR827 slicing target gi|691466862 at nt 517 for category 1. **c** Example of cgi-miR396 slicing target gi|691443742 at nt 931 for category 1. **d** Example of cgi-mir169 slicing target gi|691493380 at nt 1312 for category 2

**Fig. 6:**
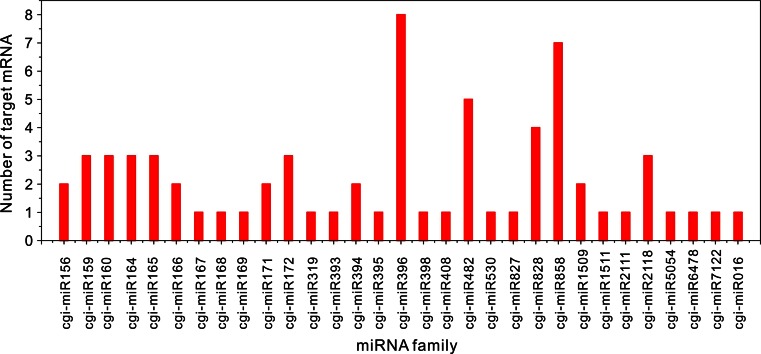
Number of targets for each miRNA family. Cgi-miR016 is novel miRNA, the others are known miRNAs

To further investigate the regulatory functions of miRNAs, degradome sequencing for samples of young roots, tender shoots, young leaves and flower buds from *C. gigantea* produce 19,967,565 raw reads and 9,664,154 unique reads. Then, 9,193,054 unique reads could be matched with 43,648 *C. gigantea* mRNAs using BLASTN program (Table 2). Finally, the targets were grouped into three categories according to relative abundance of degradome reads mapping at the predicted miRNA target site relative to the abundance of the reads located at other sites. In category 0, the peak value of tags was found at the predicted cleavage site of miRNA and there was only one maximum on the transcript. If the abundance of tags was between the median and the maximum, it was grouped as category 1. In category 2, the abundance of tags was equal to, or less than the median (Fig. 5). Thus, a total of 60 miRNA targets from 95 miRNAs in 30 known miRNA families and one novel miRNA target were identified (Table 3; Fig. 6). Also, a total of 169 miRNAs and target pairs were obtained, including 111, 14 and 45 pairs belonging to Categories 0, 1 and 2, respectively. The differences in the abundance of degradome reads at various targets were large (ranging from 5 to 853), suggesting that distinct miRNAs had various cleavage abilities. Moreover, multiple targets might be regulated by one miRNA family, and multiple miRNA families might target the same gene. For example, the cgi-mi396 and cgi-mi828 families regulated 8 and 4 target genes, respectively, while the cgi-miR165 and cgi-miR166 families regulated the same target gene.

**Table 2:** Summary of data from *Cercis gigantea* degradome sequencing

Type	Number of reads	Percentage (%)
Total unique reads	9,664,154	100
Reads mapping to the transcripts	9,193,054	95.13
Reads mapping to target site	6,916	0.072
Total Number of input cDNAs	77,024	100
Number of covered cDNAs	43,648	56.65

**Table 3:** Targets of *Cercis gigantea* miRNA identified by degradome sequencing

MiRNA family	Target_gi	Cleavage site	Category	Raw reads	Putative function
cgi-miR156	691448577	1810	2	6	Squamosa promoter-binding-like protein
	691462079	1602	0	130	Squamosa promoter-binding-like protein
cgi-miR159	691490413	545	2	11	Hypothetical protein PHAVU
	691477367	1209	0	22	MYB transcription factor
	691451897	2559	0	50	Putative protein
cgi-miR160	691466148	799	0	431	Auxin response factor
	691465468	1585	0	664	Auxin response factor
	691465470	2232	0	664	Auxin response factor
cgi-miR164	691460139	761	0	309	Domain-containing protein
	691447416	2079	2	8	T4P13.15 protein
	691452741	1647	2	5	Transport protein Sec61 subunit alpha-like isoform X1
cgi-miR165	691442095	2327	0	385	Homeobox-leucine zipper protein
	691442087	624	0	385	Homeobox-leucine zipper protein
cgi-miR166	691442095	2327	0	385	Homeobox-leucine zipper protein
	691442087	624	0	385	Homeobox-leucine zipper protein
cgi-miR167	691459251	3407	0	57	Auxin response factor
cgi-miR168	691449279	529	0	248	Protein argonaute 1
cgi-miR169	691493380	1312	2	12	Nuclear transcription factor
	691480362	131	0	16	Unknown
cgi-miR171	691465196	12	2	11	Scarecrow-like protein
	691459148	1657	0	14	Scarecrow-like protein
cgi-miR172	691479449	1502	0	15	Ethylene-responsive transcription factor
	691450023	867	2	25	Glutathione S-transferase U25
	691490548	605	2	5	Translation factor SUI1 homolog
cgi-miR319	691451897	2559	0	50	Putative protein
cgi-miR393	691475213	1794	0	424	Transport inhibitor response
cgi-miR394	691463334	1603	2	19	Emb|CAB89363.1
	691457013	1220	1	27	F-box only protein
cgi-miR395	691479675	242	2	7	Sulfate transporter
cgi-miR396	691424744	621	0	6	Unknown
	691488591	234	0	18	Hypothetical protein
	691443742	931	1	12	Short chain alcohol dehydrogenase
	691455724	1171	2	5	Ultimate buster-like protein
	691461032	876	0	41	Growth-regulating factor
	691480372	1486	2	6	Pentatricopeptide repeat-containing protein
	691480627	821	0	72	Hypersensitive-induced response protein
	691485626	487	0	145	Growth-regulating factor
	691488591	234	0	18	Protein PHAVU_001G141400 g
cgi-miR398	691487022	775	2	5	Transcription factor
cgi-miR408	691492924	539	2	13	Naringenin,2-oxoglutarate 3-dioxygenase
	691492802	82	0	853	Basic blue protein
cgi-miR482	691492924	539	2	13	Naringenin,2-oxoglutarate 3-dioxygenase
	691497201	521	0	25	Ribonuclease H protein
	691435294	757	2	7	TIR-NBS-LRR class disease resistance protein
	691457093	2200	0	107	Serine/threonine-protein phosphatase
	691435270	833	0	47	Disease resistance protein RPM1
cgi-miR530	691451139	279	0	55	Uncharacterized protein
cgi-miR827	691466862	517	1	15	SPX domain-containing membrane protein
cgi-miR828	691513396	29	0	38	MYB transcription factor
	691488014	432	0	28	Transcription factor WER-like isoform X1
	691486325	194	0	17	Transcription factor
	691469139	502	0	50	Transcription factor MYB23
cgi-miR858	691496673	458	0	13	Myb-related transcription factor
	691484799	338	0	17	Transcription factor MYB76
	691469139	468	1	12	Transcription factor MYB23
	691477366	359	2	5	Hypothetical protein POPTR_0003s06320g
	691478191	396	0	54	Transcription factor TT2
	691484368	399	0	84	Transcription factor MYB29
	691496673	458	0	13	Myb-related transcription factor
cgi-miR1509	691490282	253	2	5	Unknown
	691444031	476	0	26	Unknown
cgi-miR1511	691470779	431	0	31	Unknown
cgi-miR2111	691466946	1110	0	86	Transcription factor
cgi-miR2118	691435270	833	0	47	Disease resistance protein RPM1
	691465334	677	2	6	Zinc transporter
	691457093	2200	0	107	Serine/threonine-protein phosphatase
cgi-miR5054	691474734	368	2	9	Emb|CAB72159.1
cgi-miR6478	691455866	1598	2	6	U-box domain-containing protein
cgi-miR7122	691490282	401	0	18	Unknown
cgi-miR016	691475785	627	1	11	Putative protein

According to the best BLASTX hits from the alignments using soybean and *Arabidopsis thaliana* protein sequences as references, putative target gene names and ‘CDS’ (coding DNA sequences) were determined, then Blast2GO (Conesa et al. 2005) software was used to predict GO terms. Based on the predicted GO annotations of targets regulated by known *C. gigantea* miRNAs, 28.3 % of miRNA targets (17) were found to be transcription factors (Table 3). These results are consistent with other reports (Wu et al. 2006; Yang et al. 2006). All putative targets regulated by the miR167 family were plant-specific auxin response factors (ARFs) participating in hormone signal transduction (Wu et al. 2006). The ARFs bound specifically to the TGTCTC sequence and regulated auxin response, indicating that the cgi-miR167 family might play a key role in regulating the growth process of *C. gigantea*. The miR482 family targeted the TIR-NBS-LRR disease resistance, gene family implying that cgi-miR482 might be involved in responses to disease in *C. gigantea* (Yang et al. 2015). Furthermore, the enrichment of the targets was found to be involved in the auxin-mediated signaling pathway, regulation of transcription, formation patterns of xylem and phloem, and root hair cell differentiation (Table S3–4). For instance, the targets of cgi-miR160, cgi-miR393 and cig-miR396 families were involved in the auxin-mediated signaling pathway; the targets of other three miRNA familes including cgi-miR165, cgi-miR168 and cgi-miR828 were involved in root hair cell differentiation.

### Conserved and non-conserved miRNAs

Recent studies revealed that the majority of miRNAs were conserved across the plant species and involved in various processes such as growth and development as well as stress tolerance (Xie et al. 2010). However, some miRNAs were just identified in a few plant species (Jones-Rhoades et al. 2006). According to the definition of conserved and non-conserved miRNAs (see Materials and methods), there were 27, 18, 7 and 23 *C. gigantea* miRNA families belonging to Classes I, II, III, and IV, respectively (Fig. S4a).

Based on the comparisons of different conservations among the four classes of miRNA families, the average miRNA expression of Class I was 14.26 times that of Class II, 10.43 times that of Class III, and 222.41 times that of Class IV (Fig. S4b). The miRNAs with high expression were mainly included in the Class I, for example, the top five miRNAs with highest expressions (the count of tags were 564,107, 61,276, 37,106, 11,146 and 10,005, larger than 10,000), which belonged to the miR166, miR166, miR159, miR482, and miR168 family, come from Class I. On the other hand, the non-conserved miRNA families were found to be not only expressed at lower level than Class I, but also contained a smaller number of family members and targets (Fig. S4).

Furthermore, the targets regulated by miRNAs were grouped into two categories according to the conservation level of miRNA families: (a) conserved target, which was regulated by the highly conserved miRNA family; (b) non-conserved target that was regulated by non-conserved miRNA families (Class II–IV). 46 out of a total of 61 targets detected in this study (75.4 %) were conserved targets, accounting for a large fraction of targets. What is more, most conserved targets are found to be transcription factors but many non-conserved targets are likely to be diverse genes that play roles in a broad range of specific biological processes such as root hair cell differentiation (Table S3). A comparison of the size as well as cleavage site positions for two categories of targets showed that the average size of the conserved target was significantly larger than that of the non-conserved target. Besides, the cleavage sites of 25 conserved targets (54.3 %) were located on the last ½ of the gene, whereas 86.7 % of the non-conserved targets (13) had the cleavage sites located on the first ½ of the gene (Fig. S5). These findings suggest that the highly conserved miRNA tended to target on the last ½ of gene whereas the non-conserved miRNA preferred to target on the first ½ of the gene.

We also studied the evolutionary pattern of two categories of miRNA targets. These results of BLASTX searches showed that 89.1 % of conserved targets had orthologs in soybeans, while there were no orthologs discovered for 66.7 % of non-conserved target genes (Table S5). After aligning the homologous sequences, the average nucleotide diversity within the conserved target genes (0.156) was significantly lower than that within non-conserved target genes (0.012), suggesting that non-conserved target genes might evolve faster than conserved target genes. Although the average Ka/Ks ratio in the non-conserved target genes was 0.21, which was little lower than that of the conserved target genes (0.27); however, 33.3 % of the non-conserved target genes have too many differences with their corresponding orthologs to calculate Ka/Ks ratio. Therefore, both categories of target genes were considered to be under strong negative selection but experienced different evolutionary processes.

## Discussion

Regulation of gene expression guided by miRNAs has been reported in many plants, such as *Arabidopsis thaliana* (Addo-Quaye et al. 2008), rice (Li et al. 2010), and other plants (Xu et al. 2013; Zhang et al. 2014). However, *C. gigantea* miRNAs and their targets remain unknown. Recently, high-throughput sequencing technology provides an efficient, convenient and credible way to investigate miRNAs and their regulatory functions. In this study, we studied *C. gigantea* miRNAs and their targets using high-throughput sequencing and degradome analysis.

The sRNA length analysis of *C. gigantea* showed that the 21-nt and 24-nt redundant sRNAs displayed the highest redundancies, whereas the 24-nt unique sRNA was the most abundant (50.8 %, see Fig. 1). Similar results were found in other species, such as *Populus balsamifera* (Morin et al. 2008) and *Vriesea carinata* (Guzman et al. 2013). Vitantonio et al. (2010) reported similar results and conclusions. The bases at 5′end of miRNA were expected to be U, which favored combination with Argonaute 1 (Mi et al. 2008; Takeda et al. 2008). In the present study, we also found that the bases at 5′end of most miRNAs (84.4 %) were U. Liang et al. (2011) and Xu et al. (2012) reported that various miRNAs had different expression levels. Similarly, we identified 194 known miRNAs and 23 novel miRNAs with large variation in miRNA expression in the current study (Table 2), and the abundance of most miRNAs (85 %) is more than 5. In accordance with the studies conducted by Gonzalez-Ibeas et al. (2011) and Puzey et al. (2012), the number of miRNA family members varied greatly, ranging from 1 (miR157, miR394 and miR399) to 20 (miR166), as shown in Fig. 2. Moreover, the predicted annotations of most target genes accorded with biological functions of genes in *Arabidopsis thaliana* (Fahlgren et al. 2007), *Medicago sativa* Linn (Szittya et al. 2008) and rice (Li et al. 2010). In short, although some miRNAs might be not included in our database since the strict criteria were used to identify novel miRNAs from trancriptome sequence, our results of *C. gigantea* sRNA length distribution, and the base preference, length distribution and expression of miRNAs, as well as the functions of target genes regulated by miRNA were completely consistent with previous findings, suggesting that the high-throughput sequencing and degradome analysis for *C. gigantea* miRNAs and their targets are reliable.

In this study, highly conserved miRNA families (Class I) have higher level of expression and more abundant types of target genes mainly involved in regulation of transcription and other basic life processes during plant growth and development compared to the non-conserved miRNAs (Table S4; Fig. S4). For example, the homeobox-leucine zipper protein regulated by miR166 is involved in leaf morphogenesis, regulation of vascular development and lateral organ polarity, and formation of the meristem (Singh et al. 2014). The target of miR159 was a transcription factor (Yang et al. 2014); the miR168 targeted Argonaute 1 protein (Liang et al. 2013), which played important roles in recognition of the target mRNA first and then degrading or repressing its translation in the nucleus (Li et al. 2010).

On the other hand, the miRNA expression level and the number of miRNA targets in the non-conserved miRNA families (Class II–IV) were low, and the enrichments of their targets were just involved in the differentiation of root hair cells. A wheat miRNA study conducted by Yao et al. (2007) showed that novel miRNAs were generally considered to be evolutionarily young, species-specific and having specific functions. The expression level of the novel miRNAs was usually found to be lower than the known miRNAs (Allen et al. 2004; Fahlgren et al. 2007). Similar results were found in our study (Table S1–2). In addition, the miRNAs* (the complementary strands of functional mature miRNAs) still expressed at lower level than their corresponding miRNAs, which were in accordance with the consequence of the rapid degradation of the miRNA* chain during the formation of mature miRNA (Ding et al. 2009; Liu et al. 2014). Similarly, the targets regulated by these miRNAs were involved in different process from highly conserved miRNA targets (Table S3–4). For instance, the targets of Class II miRNAs were mainly involved in the differentiation of root hair cells, which could be easily explained by the specific appearance of miRNAs from Class II in dicotyledons, while dicotyledons which generally had straight roots were different from monocotyledons having fibrous root systems. Therefore, it is easy to understand the reason why the miRNAs that regulate the root development were dicotyledon only.

Why are there many differences between highly conserved miRNAs and non-conserved miRNAs in *C. gigantea*? Generally, plant miRNAs were found to form by inverted duplication events resulting in a high proportion of complementary nucleotides to the parental locus, having ability to produce small RNA targeting the parental transcript when expressed (Allen et al. 2004; Fahlgren et al. 2007; Axtell and Bowman 2008). They evolved neutrally (Axtell et al. 2007; Chen and Rajewsky 2007). Several researches also suggested that highly conserved miRNAs families expanded and specialized by duplication and sub- or neofunctionalization in a long time due to their participation in important processes during plant growth and development (Maher et al. 2006; Chen and Rajewsky 2007; Rubio-Somoza et al. 2009), whereas most non-conserved miRNAs were considered to be evolutionarily transient loci which were born frequently but were also lost frequently, going through birth-and-death process (Fahlgren et al. 2007; Axtell and Bowman 2008; Lu et al. 2008a). In such cases, non-conserved miRNAs were supposed to be evolutionarily young with the characteristics of lower expression level, fewer family members and targets (Axtell et al. 2007; Fahlgren et al. 2007, 2010). Similar results were found in the current study (Fig. S4). In addition, Kutter et al. (2007) and Fahlgren et al. (2010) pointed out that some non-conserved miRNAs were kept in the population for a long time due to their special function. The type of ‘old’ non-conserved miRNAs was found in *C. gigantea* as well. In fact, some Classe II miRNAs that regulate targets involved in the development of root hairs should exist prior to the differentiation of monocotyledons and dicotyledons. In a word, the different characteristics between highly conserved miRNAs and non-conserved miRNAs in *C.gigantea* were consistent with neutral evolution model.

### *Author contribution statement*

WG and YZ conceived and designed the study, carried out data analysis, interpreted the entire results and drafted the manuscript. QW carried out data analysis and drafted the manuscript. YPZ and GHZ carried out data analysis and helped to draft the manuscript. QY and LCZ participated in the design of the study, interpreted the results. All authors read and approved the final manuscript.

## Electronic supplementary material

Supplementary material 1 (JPEG 412 kb)

Supplementary material 2 (JPEG 829 kb)

Supplementary material 3 (JPEG 1125 kb)

Supplementary material 4 (JPEG 692 kb)

Supplementary material 5 (JPEG 271 kb)

Supplementary material 6 (DOCX 21 kb)

Supplementary material 7 (DOCX 14 kb)

Supplementary material 8 (DOCX 12 kb)

Supplementary material 9 (DOCX 12 kb)

Supplementary material 10 (DOCX 17 kb)
