# Supplementary figures and images for: High-throughput sequencing and degradome analysis reveal neutral evolution of Cercis gigantea microRNAs and their targets
Source: Planta. 2015 Sep 5;243:83–95. doi: 10.1007/s00425-015-2389-y (PMC4698290; doi:10.1007/s00425-015-2389-y)

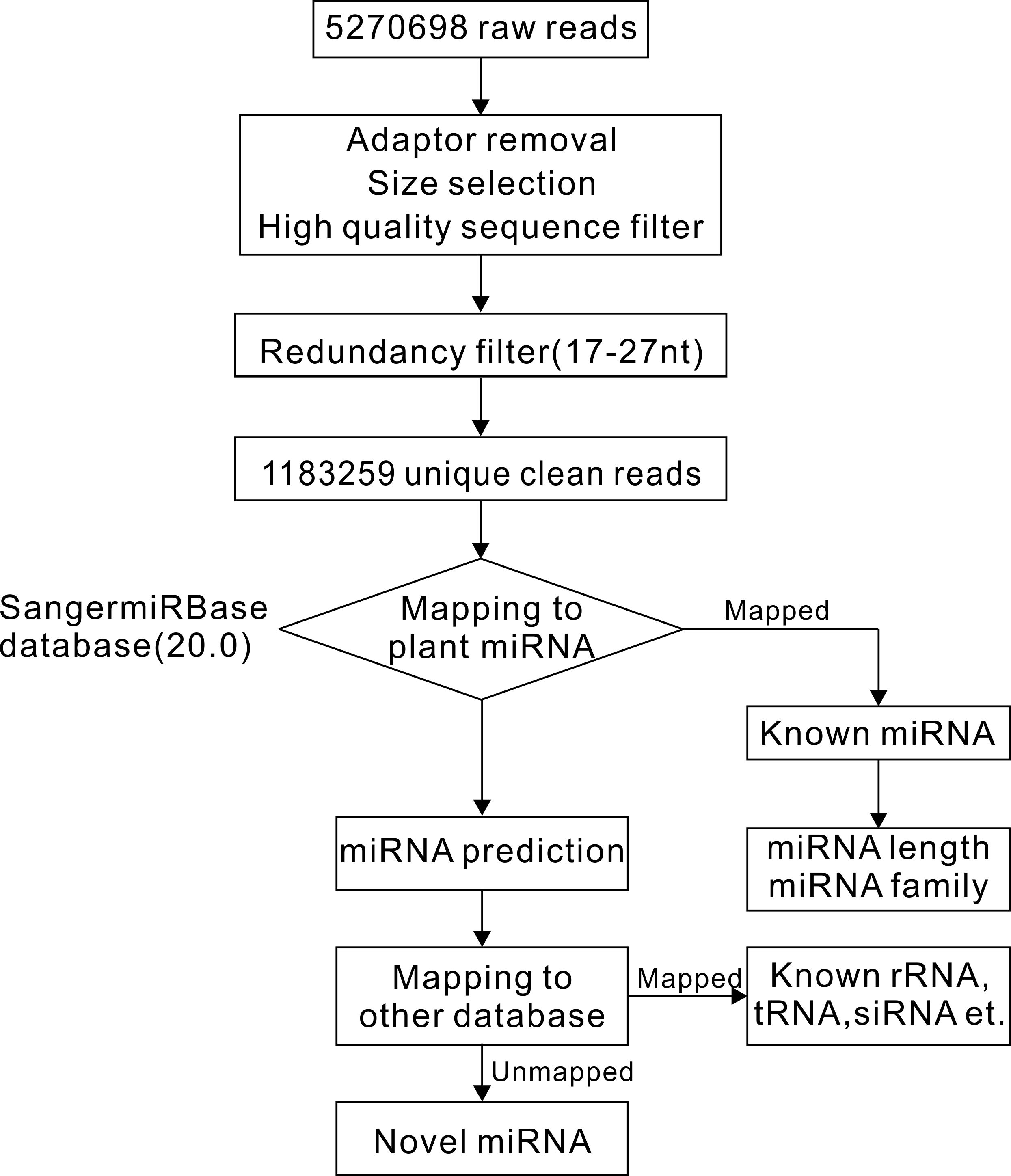

Supplement: Supplementary file 1 — Supplementary material 1 (JPEG 412 kb) [file 425_2015_2389_MOESM1_ESM.jpg]

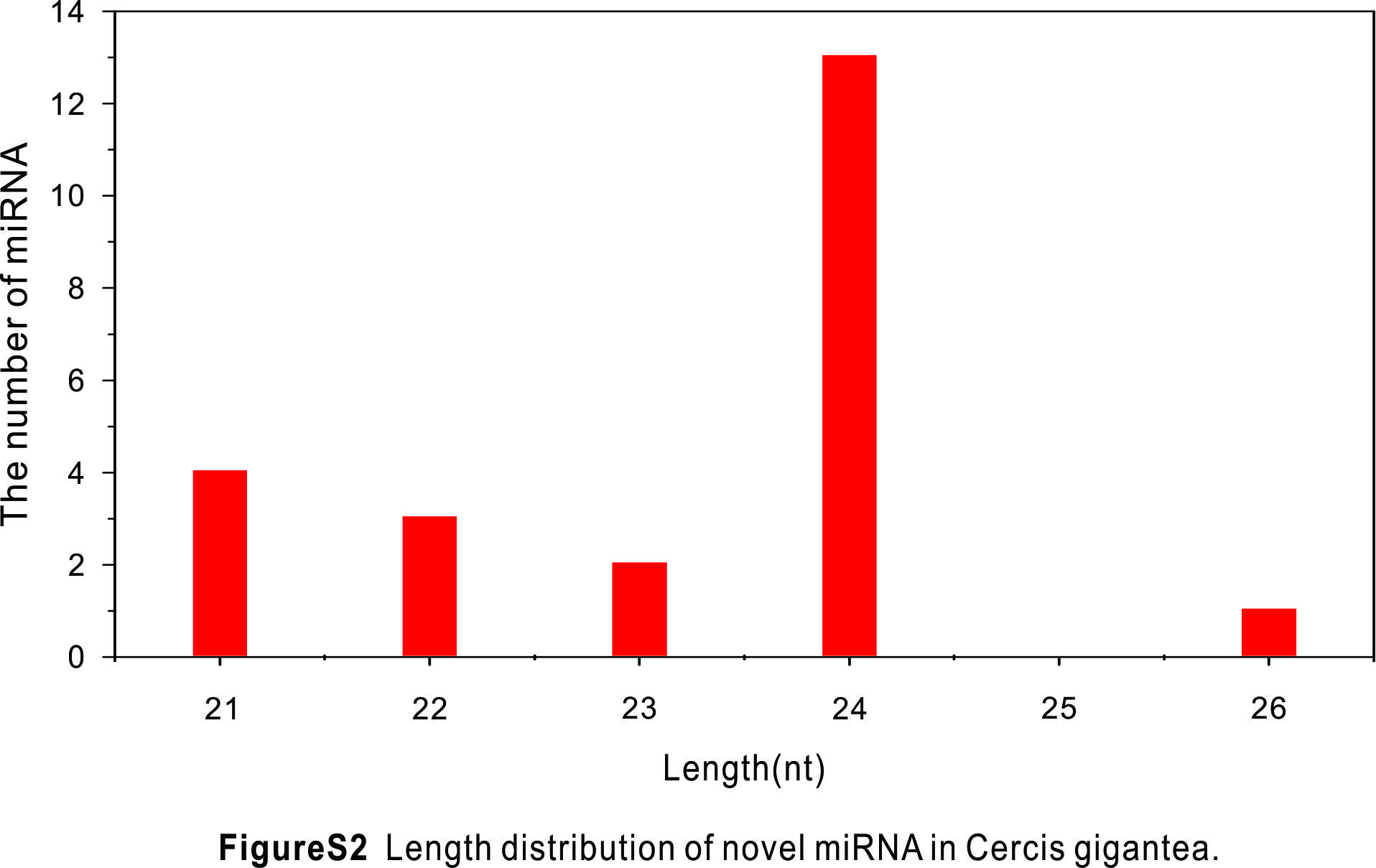

Supplement: Supplementary file 2 — Supplementary material 2 (JPEG 829 kb) [file 425_2015_2389_MOESM2_ESM.jpg]

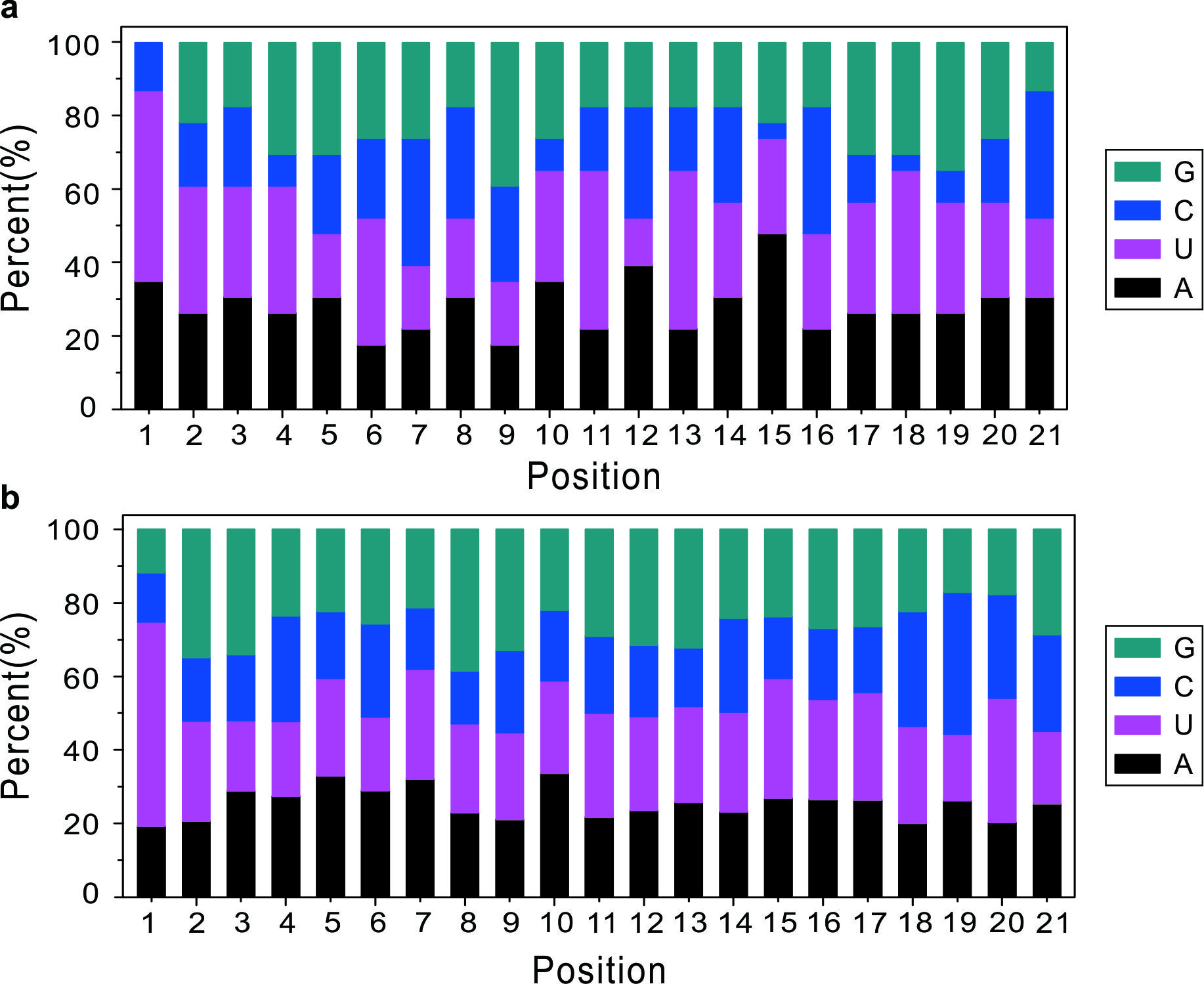

Supplement: Supplementary file 3 — Supplementary material 3 (JPEG 1125 kb) [file 425_2015_2389_MOESM3_ESM.jpg]

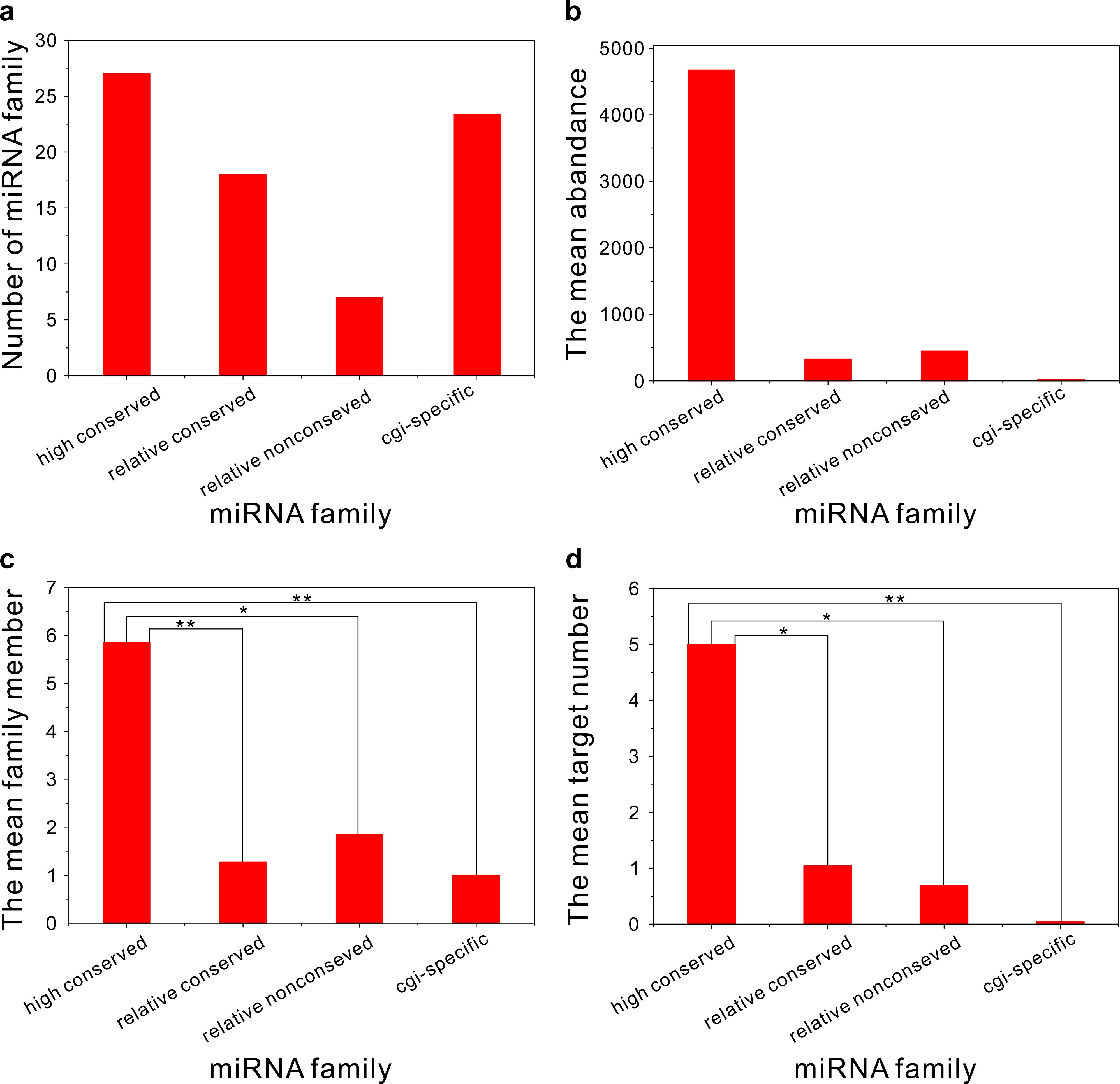

Supplement: Supplementary file 4 — Supplementary material 4 (JPEG 692 kb) [file 425_2015_2389_MOESM4_ESM.jpg]

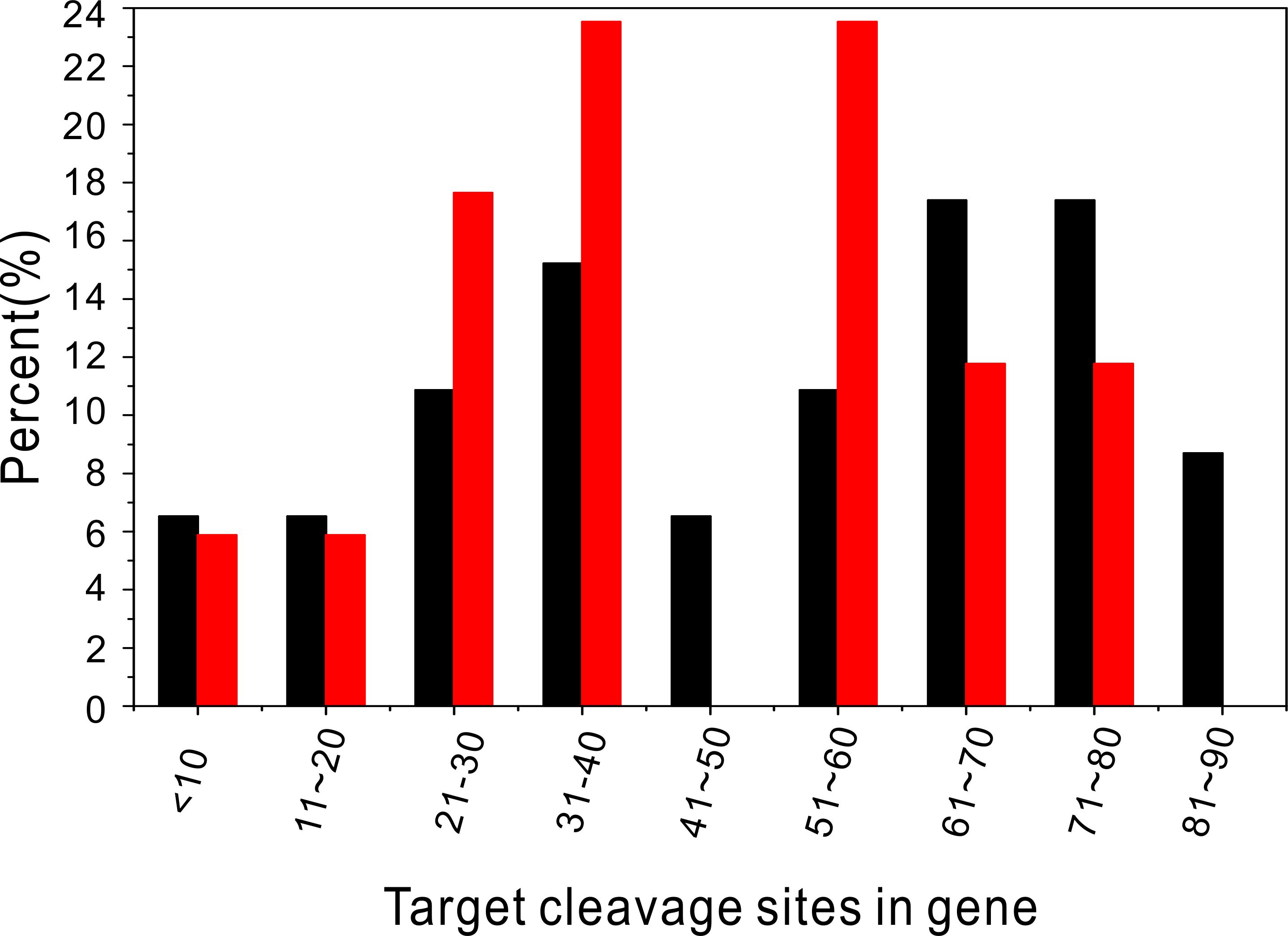

Supplement: Supplementary file 5 — Supplementary material 5 (JPEG 271 kb) [file 425_2015_2389_MOESM5_ESM.jpg]
