# Supplementary material for: High-throughput sequencing and degradome analysis reveal neutral evolution of Cercis gigantea microRNAs and their targets
Source: Planta. 2015 Sep 5;243:83–95. doi: 10.1007/s00425-015-2389-y (PMC4698290; doi:10.1007/s00425-015-2389-y)
Supplement: Supplementary file 6 — Supplementary material 6 (DOCX 21 kb) [file 425_2015_2389_MOESM6_ESM.docx]

**Table S1** Known miRNAs in *Cercis gigantea* identified by sRNA sequencing

| MiRNA family | Sequence | Length | MiRNA abundance |
| --- | --- | --- | --- |
| cgi-miR156 | TGACAGAAGAGAGTGAGCAC | 20 | 67 |
|  | TGACAGAAGAGAGTGAGCACA | 21 | 46 |
|  | TTGACAGAAGATAGAGAGCAC | 21 | 278 |
|  | TTGACAGAAGAGAGAGAGCAC | 21 | 27 |
|  | TTGACAGAAGAGAGTGAGCAC | 21 | 2 |
|  | TGACAGAAGAGAGTGAGCACT | 21 | 5 |
|  | CTGACAGAAGATAGAGAGCAT | 21 | 13 |
|  | CTGACAGAAGATAGAGAGCCT | 21 | 13 |
|  | TTGACAGAAGAGAGGGAGCAC | 21 | 3 |
|  | TGCTCACTTCTCTTTCTGTCAGC | 23 | 146 |
|  | CTGACAGAAGATAGAGAGCCC | 21 | 168 |
| cgi-miR157 | GCTCTCTATTCTTCTGTCATC | 21 | 2 |
| cgi-miR159 | TTTGGATTGAAGGGAGCTCTA | 21 | 37,106 |
|  | GAGCTCCTTGAAGTCCAATAG | 21 | 15 |
|  | AGCTGCTGAGTTATGGATCCC | 21 | 57 |
|  | GAGCTCCTTGAAGTCCAATA | 20 | 8 |
|  | TTTGGATTGAAGGGAGCTCTT | 21 | 246 |
|  | TTTGGATTGAAGGGAGCTCTA | 21 | 101 |
|  | TTTGGATTGAAGGGAGCTCCA | 21 | 101 |
|  | GAGCTCCTTGAAGTCCAATAGT | 22 | 8 |
|  | CTTGCATATCTCAGGAGCTTG | 21 | 8 |
|  | TTGGACTGAAGGGAGCTCCA | 20 | 11 |
|  | TTTGGATTGAAGGGAGCTTTTC | 22 | 8 |
| cgi-miR160 | GCGTATGAGGAGCCAAGCATA | 21 | 25 |
|  | TGCCTGGCTCCCTGTATGCCA | 21 | 311 |
|  | TGCCTGGCTCCCTGAATGCCA | 21 | 5 |
|  | TGCCTGGCTCCCTGTATGCC | 20 | 94 |
| cgi-miR162 | TCGATAAACCTCTGCATCCA | 20 | 108 |
|  | TCGATAAACCTCTGCATCCAG | 21 | 153 |
|  | GGACGCAGCGGTTCATCGATC | 21 | 15 |
| cgi-miR164 | TGGAGAAGCAGGGCACGTGCA | 21 | 1,208 |
|  | TGGAGAAGCAGGGCACGTGC | 20 | 80 |
|  | CATGTGCCCGTCTTCCCCATC | 21 | 68 |
|  | CACGTGCTCCCCTTCTCCAAC | 21 | 9 |
|  | TGGAGAAGCAGGGCACGTGCG | 21 | 2 |
|  | TGGAGAAGCAGGGCACGTTT | 20 | 2 |
|  | TGGAGAAGCAGGGCACATGCT | 21 | 2 |
| cgi-miR165 | TCGGACCAGGCTTCATCCCCC | 21 | 272 |
|  | TCGGACCAGGCTTCTTCCCC | 20 | 23 |
| cgi-miR166 | GGAATGTTGTCTGGCTCGAGG | 21 | 1,035 |
|  | CCGGACCAGGCTTCATTCCCC | 21 | 186 |
|  | TCGGACCAGGCTTCATTCCC | 20 | 490 |
|  | TCGGACCAGGCTTCATTCCCA | 21 | 2,427 |
|  | TCTCGGACCAGGCTTCATTTT | 21 | 67 |
|  | TCCGGACCAGGCTTCATTCCCC | 22 | 5 |
|  | CCTCGGACCAGGCTTCATTCCCC | 23 | 18 |
|  | TCGGACCAGGCTTCATTCCCT | 21 | 1,845 |
|  | TCTCGGACCAGGCTTCATTC | 20 | 241 |
|  | TTGGACCAGGCTTCATTCCCC | 21 | 217 |
|  | TTCGGACCAGGCTTCATTCCCC | 22 | 5,595 |
|  | TCGGACCAGGCTTCATTCCCC | 21 | 564,107 |
|  | GGAATGTTGGCTGGCTCGAGG | 21 | 2,180 |
|  | TCTCGGACCAGGCTTCATTCC | 21 | 61,276 |
|  | GGAATGTTGTTTGGCTCGAGG | 21 | 978 |
|  | TCGGACCAGGCTTCATTCCCG | 21 | 30 |
|  | AATGAGGTTTGATCCAAGATC | 21 | 2 |
|  | TCGGACCAGGCTTCATTTCCC | 21 | 196 |
|  | CGGACCAGGCTTCATTCCCC | 20 | 1,287 |
|  | TCGAACCAGGCTTCATTCCCC | 21 | 258 |
| cgi-miR167 | TGAAGCTGCCAGCATGATCTA | 21 | 54 |
|  | GGTCATGCTGTGACAGCCTCACT | 23 | 6 |
|  | TGAAGCTGCCAGCATGATCTG | 21 | 7 |
|  | GATCATGCGGCAGTTTCACC | 20 | 5 |
|  | AGGTCATCTTGCAGCTTCAAT | 21 | 4 |
|  | TGAAGCTGCCAGCATGATCTT | 21 | 36 |
|  | TGAAGCTGCCAGCATGATCTGA | 22 | 66 |
|  | TGAAGCTGCCAGCATGATCTGG | 22 | 167 |
|  | TGAAGCTGCCAGCATGATCTC | 21 | 25 |
|  | GGTCATGCTCTGACAGCCTCACT | 23 | 1 |
|  | GATCATGTTCGCAGTTTCACC | 21 | 1 |
| cgi-miR168 | TCGCTTGGTGCAGGTCGGGAA | 21 | 10,005 |
|  | CCCGCCTTGCATCAACTGAAT | 21 | 1,390 |
|  | TCGCTTGGTGCAGGTCGGGA | 20 | 24 |
|  | CGCTTGGTGCAGGTCGGGAA | 20 | 26 |
|  | TCGCTTGGTGCAGGTCGGGAACT | 23 | 11 |
|  | TCGCTTGGTGCAGGTCGGG | 19 | 6 |
|  | TCGCTTGGTGCAGGTCGGGAAT | 22 | 102 |
|  | TCGCTTGGTGCAGATCGGGAC | 21 | 13 |
|  | CCCGCCTTGCATCAACTGAATT | 22 | 140 |
| cgi-miR169 | CAGCCAAGGATGACTTGCCGG | 21 | 3 |
|  | TGAGCCAAGGATGACTTGCCGG | 22 | 5 |
|  | AGCCAAGGATGACTTGCCGG | 20 | 3 |
|  | CAGCCAAGAATGACTTGCCGG | 21 | 13 |
|  | CTTGGCTATATTGGGCTCTCT | 21 | 39 |
| cgi-miR171 | CGAGCCGAATCAATATCACTC | 21 | 6 |
|  | TTGAGCCGTGCCAATATCACA | 21 | 25 |
|  | TGATTGAGCCGTGCCAATATC | 21 | 75 |
|  | TTGAGCCGTGCCAATATCACG | 21 | 6 |
|  | TATTGGCCTGGTTCACTCAGA | 21 | 103 |
|  | TTGAGCCGCGCCAATATCACT | 21 | 13 |
|  | TTGAGCCGCGTCAATATCTTA | 21 | 9 |
|  | TATTGGCCTGGCTCACTCAGA | 21 | 103 |
| cgi-miR172 | GTAGCATCATCAAGATTCACA | 21 | 7 |
|  | AGAATCTTGATGATGCTGCAT | 21 | 334 |
|  | AGAATCTTGATGATGCTGCAG | 21 | 19 |
|  | GGAGCATCATCAAGATTCACA | 21 | 17 |
|  | GCAGCAGCATCAAGATTCACA | 21 | 13 |
|  | TGAATCTTGATGATGCTGCAT | 21 | 1 |
|  | CGATGTTGGTGAGGTTCAATC | 21 | 4 |
|  | CGAATCTTGATGATGCTGCAT | 21 | 1 |
| cgi-miR319 | TTGGACTGAAGGGAGCTCCC | 20 | 6,702 |
|  | TTTGGACTGAAGGGAGCTCCT | 21 | 281 |
|  | TCTTGGACTGAAGGGAGCTCC | 21 | 256 |
|  | ATTGGATTGAAGGGAGCTCCC | 21 | 47 |
|  | TTGGACTGAAGGGAGCTCCCT | 21 | 1,566 |
|  | TAGCTGCCGACTCATTCATCCA | 22 | 8 |
|  | TTGGACTGAAGGGAGCTCCTTC | 22 | 208 |
|  | TTGGACTGAAGGGAGCTCCT | 20 | 91 |
|  | TTGGACTGAAGGGAGCTCCCT | 21 | 1,914 |
|  | CTTGGACTGAAGGGAGCTCCC | 21 | 6,420 |
|  | TTGGACTGAAGGGAGCTCCCC | 21 | 74 |
|  | TTGGACTGAAGGGAGCTCCCA | 21 | 79 |
| cgi-miR390 | CGCTATCCATCCTGAGTTTC | 20 | 18 |
|  | AAGCTCAGGAGGGATAGCGCC | 21 | 1,151 |
|  | AGCTCAGGAGGGATAGCGCC | 20 | 17 |
|  | CGCTATCCATCCTGAGTTTCA | 21 | 37 |
|  | TAAGAAGAATAAGCTCAGGAT | 21 | 10 |
| cgi-miR393 | TCCAAAGGGATCGCATTGATC | 21 | 28 |
|  | TCCAAAGGGATCGCATTGATCC | 22 | 15 |
|  | ATCATGCTATCCCTTTGGATT | 21 | 7 |
|  | TTCCAAAGGGATCGCATTGATC | 22 | 187 |
|  | TCATGCGATCCCTTAGGAATT | 21 | 18 |
|  | TTTGGATTCCTCCTTTGGTGG | 21 | 6 |
| cgi-miR394 | TTGGCATTCTGTCCACCTCC | 20 | 76 |
| cgi-miR395 | CTGAAGTGTTTGGGGGAACTC | 21 | 3 |
|  | TGAAGTGTTTGGGGGAACTCC | 21 | 15 |
|  | AGTTCCTCTGAACACTTCATA | 21 | 1 |
|  | AGTTCCTCTGAACGCTTCATA | 21 | 2 |
|  | TGAAGTGTTTGGGGGAACTCC | 21 | 19 |
| cgi-miR396 | TTCAATAAAGCTGTGGGAGG | 20 | 146 |
|  | TTCCACAGTTTTCTTGAACTG | 21 | 3 |
|  | TTCCACAGCTTTCTTGAACTG | 21 | 4,330 |
|  | TTCCACAGCTTTCTTGAACTTTC | 23 | 10 |
|  | TTCCACAGCTTTCTTGAACTTT | 22 | 65 |
|  | GCTCAAGAAAGCTGTGGGACA | 21 | 30 |
|  | TTCCACAGCTTTCTTGAACTT | 21 | 1,235 |
|  | TTCCACAGCTTTCTTGAACTGT | 22 | 4 |
|  | TCCACAGCTTTCTTGAACTG | 20 | 2 |
|  | TTCCACAGCTTTCTTGAACTA | 21 | 36 |
|  | GTTCAATAAAGCTGTGGGAGG | 21 | 1,674 |
|  | TCCACAGGCTTTCTTGAACTG | 21 | 1 |
| cgi-miR398 | TGTGTTCTCAGGTCACCCCTT | 21 | 5 |
|  | TGTGTTCTCAGGTCGCCCCTG | 21 | 39 |
| cgi-miR399 | TGCCAAAGGAGAGTTGCCCTG | 21 | 2 |
| cgi-miR403 | TTAGATTCACGCACAAACTTG | 21 | 78 |
|  | TTAGATTCACGCACAAACTCG | 21 | 3 |
| cgi-miR408 | ATGCACTGCCTCTTCCCTGGC | 21 | 47 |
|  | TGCACTGCCTCTTCCCTGGCTC | 22 | 74 |
|  | TGCACTGCCTCTTCCCTGGC | 20 | 36 |
|  | CAGGGAAGAGACAGAGCATGG | 21 | 78 |
| cgi-miR477 | ACTCTCCCTCAAGGGCTTCCG | 21 | 33 |
| cgi-miR479 | TGTGGTATTGGTTCGGCTCATC | 22 | 105 |
| cgi-miR482 | TGGGAATGGGCTGTTTGGGAAG | 22 | 3 |
|  | TCTTCCCTACACCTCCCATACC | 22 | 11,146 |
|  | GGAATGGGCTGTTTGGGAAGT | 21 | 8 |
|  | TCTTTCCAACTCCTCCCATACC | 22 | 4 |
|  | TTTCCAATTCCACCCATTCCTA | 22 | 39 |
|  | GGAATGGGCTGTTTGGGAAGA | 21 | 3,628 |
|  | TGCATTTGCACCTGCACCTAC | 21 | 1 |
| cgi-miR530 | TGCATTTGCACCTGCACTTTA | 21 | 6 |
| cgi-miR535 | TGACAACGAGAGAGAGCACGC | 21 | 2 |
| cgi-miR827 | TTAGATGACCATCAACAAACA | 21 | 21 |
| cgi-miR828 | TCTTGCTCAAATGAGTATTCCA | 22 | 2 |
| cgi-miR858 | TTTCGTTGTCTGTTCGACCTT | 21 | 3 |
|  | TTCGTTGTCTGTTCGACCTTG | 21 | 290 |
|  | CTCGTTGTCTGTTCGACCTTG | 21 | 98 |
| cgi-miR1310 | AGGCATCGGGGGCGCAACGCCC | 22 | 19 |
| cgi-miR1448 | TCTTTCCAACGCCTCCCATACC | 22 | 5,938 |
| cgi-miR1509 | TTAATCAAGGAAATCACAGTTG | 22 | 174 |
| cgi-miR1511 | AACCAGGCTCTGATACCA | 18 | 52 |
| cgi-miR1515 | TCATTTTTGCGTGCAATGATCC | 22 | 5 |
| cgi-miR2111 | TAATCTGCATCCTGAGGTTT | 20 | 39 |
|  | GTCCTCAGAATGCAGATTACC | 21 | 46 |
| cgi-miR2118 | TTGCCGATTCCACCCATTCCT | 21 | 22 |
|  | TTGCCGATTCCACCCATTCCTA | 22 | 5,469 |
|  | GGAGATGGGAGGGTCGGTAAAG | 22 | 93 |
|  | TTACCGATTCCACCCATTCCTA | 22 | 2 |
| cgi-miR2916 | GCGGATGTTGCTTTTAGGA | 19 | 251 |
| cgi-miR3630 | TTTGGGAATCTCTCTGATGCAC | 22 | 19 |
|  | ATGGGAATCTCTCTGATGCTT | 21 | 9 |
| cgi-miR5037 | AACCCTCAAAGGCTTCCACT | 20 | 37 |
| cgi-miR5054 | CCCCACGGTGGGCGCCA | 17 | 24 |
| cgi-miR5139 | AAACCTGGCTCTGATACCA | 20 | 11 |
| cgi-miR5211 | TGTCGCAGGAGCGTTGGCACC | 21 | 20 |
| cgi-miR5225 | CCTGTCGTAGGAGAGATGACGC | 22 | 5 |
|  | CCTGTCGTAGGAGAGATGACGCCT | 24 | 11 |
| cgi-miR5261 | TCATTGTAGATGGCTTTGGCT | 21 | 12 |
| cgi-miR5368 | GGGACAGTCTCAGGTAGACA | 20 | 15 |
|  | CCTGGGATTGGCTTTGGGCCT | 21 | 21 |
| cgi-miR5788 | TGGATGTGACATACTCTAGTA | 21 | 1 |
| cgi-miR6173 | AGCCGTAAACGATGGATACT | 20 | 35 |
| cgi-miR6478 | CCGACCTTAGCTCAGTTGGC | 20 | 288 |
| cgi-miR7122 | TTATACAGTGAAATCACGGTCG | 22 | 1 |
| cgi-miR8155 | TAACCTGGCTCTGATACCA | 19 | 27 |
