# Supplementary material for: High-throughput sequencing and degradome analysis reveal neutral evolution of Cercis gigantea microRNAs and their targets
Source: Planta. 2015 Sep 5;243:83–95. doi: 10.1007/s00425-015-2389-y (PMC4698290; doi:10.1007/s00425-015-2389-y)
Supplement: Supplementary file 7 — Supplementary material 7 (DOCX 14 kb) [file 425_2015_2389_MOESM7_ESM.docx]

**Table S2** Novel miRNAs in *Cercis gigantea* identified by sRNA sequencing

| **Pre-miRNA** |  | **Precursor miRNAs** | | | **Mature miRNAs** |  |  | **miRNA*** |  |
| --- | --- | --- | --- | --- | --- | --- | --- | --- | --- |
|  | **mRNA name** | **length** | **MFE** | **AMFE** | **Sequence** | **Length** | **Reads** | **Sequence** | **Reads** |
| cgi-miR001 | c1121_g1_i1 | 101 | -27 | -26.73 | ACTTGGTCTAAGGTTGGGAAAGCT | 24 | 19 | GGGACCAGCGTGCGCCTTTAAGAT | 1 |
| cgi-miR002 | c5919_g1_i1 | 144 | -44.9 | -31.18 | ATTGGTCATTGAATTATTATTCTG | 24 | 54 | AGAATAATAATTCAATGACCAATT | 1 |
| cgi-miR003 | c7761_g1_i2 | 186 | -104.2 | -56.02 | CAGGACACAGGTTCGAGGGCCGGTAC | 26 | 5 | GTACCGGCCCTCGAACCTGTGTCTTG |  |
| cgi-miR004 | c8753_g2_i1 | 80 | -16.3 | -20.38 | TATGATCAGATGGCTCTTTGTT | 22 | 5 | AACTTTAGTTTACTTTTTGGCACG | 2 |
| cgi-miR005 | c12000_g1_i2 | 183 | -56.52 | -30.89 | AGACCGGACACGCCAGCAGTCACG | 24 | 6 | ND |  |
| cgi-miR006 | c12422_g1_i1 | 182 | -58.7 | -32.25 | TATGGTTCCGTACGACAAATTAGT | 24 | 6 | ATTAATTTGTCGTACGGAACCATA | 3 |
| cgi-miR007 | c18403_g1_i1 | 76 | -13.5 | -17.76 | TTAGCTCGATCTAACGGTGGGACT | 24 | 46 | CACCGTTAGATCGAGCTAAAATT | 5 |
| cgi-miR008 | c77500_g1_i1 | 72 | -16.6 | -23.06 | TTGAAACTGCTCTGACCTTGT | 21 | 5 | ACATTGCAAGTCAGGATCAGT | 1 |
| cgi-miR009 | c85120_g1_i1 | 84 | -15.4 | -18.33 | CCACCGTTCAGATTGAAGAGCT | 22 | 39 | ATTTCAGCTCGATCCAATAGTAGA | 4 |
| cgi-miR010 | c92083_g1_i1 | 72 | -19.3 | -26.81 | TGAATTAGAGAACTTTACACCT | 22 | 9 | CTATTCAGATTGGAGACTTACGCC | 1 |
| cgi-miR011 | c94072_g1_i1 | 92 | -14.7 | -15.98 | ACGTAACTCTTCAATCTGAACGGT | 24 | 42 | ATTTTAGCTCGATCTAACAGTGGG | 1 |
| cgi-miR012 | c121193_g1_i1 | 86 | -21.5 | -25 | AAATACCCCATCCAAACACAACCT | 24 | 6 | AAGTTGTGTTTGGATGAGGTATTT | 1 |
| cgi-miR013 | c219643_g1_i1 | 82 | -15.9 | -19.39 | AGGACTAATTTGTCGTATGAAACC | 24 | 21 | AATTATGGTTTCATACGACAAATT |  |
| cgi-miR014 | c225833_g1_i1 | 77 | -16.8 | -21.82 | TTCGGCGGGGAATCACAGGCACA | 23 | 7 | AGCTTTTGTTCTCTGATTTGT | 1 |
| cgi-miR015 | c242250_g1_i1 | 174 | -55.4 | -31.84 | TACGGAACCATAATTCGAGAA | 21 | 16 | CCTCAAATTATAGTTTCGTAC | 1 |
| cgi-miR016 | c262060_g1_i1 | 154 | -60.5 | -39.29 | ATTTGGCGGACAAAGTGATTACT | 23 | 17 | TAATCACTTTGTCCGCCAAAT |  |
| cgi-miR017 | c284122_g1_i1 | 151 | -47.77 | -31.64 | TTATTTGAGGACTAAAGTGACATT | 24 | 6 | ATTTTAATCTTCGAATAATCATATT | 2 |
| cgi-miR018 | c286389_g1_i1 | 105 | -22.4 | -21.33 | ACCAAAGCGTGATCATTGTTCACT | 24 | 5 | ND |  |
| cgi-miR019 | c436969_g1_i1 | 133 | -44.3 | -33.31 | TTCTTGATTCTTGTGCATTGGAAC | 24 | 10 | AAAAAGGGGTTCCAATGCACAAGA | 1 |
| cgi-miR020 | c694826_g1_i1 | 147 | -47.63 | -32.40 | TTTAGCTCGATCTAACGGTGGGAC | 24 | 13 | GTCTTATCGTTAGATCGAGCT | 1 |
| cgi-miR021 | c693695_g1_i1 | 112 | -21.6 | -19.29 | TGTACGGAGTTAGGATTACATGCC | 24 | 113 | AGGAACATGTATTATCTCTTCATA | 3 |
| cgi-miR022 | c481848_g1_i1 | 139 | -65.6 | -47.19 | CGCTATCTATCCTGAGTTTCA | 21 | 7 | AAAGCTCAGGAGGGATAGCGCC | 2 |
| cgi-miR023 | c387_g1_i4 | 133 | -48.4 | -36.39 | TAATTCGAGGACTAATTTGTC | 21 | 25 | CAACAAATTAGTCCTCGAATT | 2 |
