# Supplementary material for: High-throughput sequencing and degradome analysis reveal neutral evolution of Cercis gigantea microRNAs and their targets
Source: Planta. 2015 Sep 5;243:83–95. doi: 10.1007/s00425-015-2389-y (PMC4698290; doi:10.1007/s00425-015-2389-y)
Supplement: Supplementary file 8 — Supplementary material 8 (DOCX 12 kb) [file 425_2015_2389_MOESM8_ESM.docx]

**Table S3** Gene Ontology function enrichment analysis of conserved *Cercis gigantea* targets

| **GO ID Name** | ***P* value** | **Number** |
| --- | --- | --- |
| GO:0010051 xylem and phloem pattern formation | 0.000130949 | 3 |
| GO:0009734 auxin mediated signaling pathway | 0.000268873 | 3 |
| GO:0009855 determination of bilateral symmetry | 0.00038202 | 3 |
| GO:0051301 cell division | 0.00053759 | 3 |
| GO:0009880 embryonic pattern specification | 0.001194645 | 2 |
| GO:0045595 regulation of cell differentiation | 0.001270461 | 2 |
| GO:0006355 regulation of transcription, DNA-dependent | 0.001479601 | 5 |
| GO:0010072 primary shoot apical meristem specification | 0.001511401 | 2 |
| GO:0010073 meristem maintenance | 0.00195752 | 2 |
| GO:0048439 flower morphogenesis | 0.003013783 | 2 |
| GO:0048864 stem cell development | 0.003207792 | 1 |
| GO:0009856 pollination | 0.004807969 | 1 |
| GO:0045926 negative regulation of growth | 0.004807969 | 1 |
| GO:0048442 sepal development | 0.004807969 | 1 |
| GO:0060776 simple leaf morphogenesis | 0.004807969 | 1 |
| GO:0007155 cell adhesion | 0.005759137 | 2 |
| GO:0045010 actin nucleation | 0.006241244 | 2 |
| GO:0010928 regulation of auxin mediated signaling pathway | 0.006405673 | 1 |
| GO:0048765 root hair cell differentiation | 0.007084323 | 2 |
| GO:0080060 integument development | 0.008000907 | 1 |
| GO:0071555 cell wall organization | 0.008724267 | 2 |
| GO:0043068 positive regulation of programmed cell death | 0.009593675 | 1 |
| GO:0016036 cellular response to phosphate starvation | 0.010311039 | 2 |
| GO:0007062 sister chromatid cohesion | 0.010726323 | 2 |
| GO:0048830 adventitious root development | 0.011183979 | 1 |
| GO:0000822 inositol hexakisphosphate binding | 0.008000907 | 1 |
| GO:0010011 auxin binding | 0.008000907 | 1 |
| GO:0035198 miRNA binding | 0.008000907 | 1 |
| GO:0035197 siRNA binding | 0.009593675 | 1 |
