# Supplementary material for: High-throughput sequencing and degradome analysis reveal neutral evolution of Cercis gigantea microRNAs and their targets
Source: Planta. 2015 Sep 5;243:83–95. doi: 10.1007/s00425-015-2389-y (PMC4698290; doi:10.1007/s00425-015-2389-y)
Supplement: Supplementary file 9 — Supplementary material 9 (DOCX 12 kb) [file 425_2015_2389_MOESM9_ESM.docx]

**Table S4** Gene Ontology function enrichment analysis of nonconserved *Cercis gigantea* targets

| **GO ID Name** | ***P* value** | **Number** |
| --- | --- | --- |
| GO:0048629 trichome patterning | 9.54E-07 | 2 |
| GO:0048765 root hair cell differentiation | 1.17E-05 | 3 |
| GO:0001708 cell fate specification | 3.33E-05 | 2 |
| GO:0010091 trichome branching | 1.03E-04 | 2 |
| GO:0003700 sequence-specific DNA binding transcription factor activity | 0.001018181 | 3 |
| GO:0080060 integument development | 0.002969295 | 1 |
| GO:0015693 magnesium ion transport | 0.00415479 | 1 |
| GO:0003677 DNA binding | 0.005818618 | 3 |
| GO:0016567 protein ubiquitination | 0.007025633 | 2 |
| GO:0045893 positive regulation of transcription, DNA-dependent | 0.010837214 | 2 |
| GO:0032880 regulation of protein localization | 0.013593172 | 1 |
| GO:0055085 transmembrane transport | 0.016375747 | 2 |
| GO:0015095 magnesium ion transmembrane transporter activity | 0.004747061 | 1 |
| GO:0070696 transmembrane receptor protein serine/threonine kinase binding | 0.005930653 | 1 |
| GO:0046873 metal ion transmembrane transporter activity | 0.006521974 | 1 |
