# Supplementary material for: High-throughput sequencing and degradome analysis reveal neutral evolution of Cercis gigantea microRNAs and their targets
Source: Planta. 2015 Sep 5;243:83–95. doi: 10.1007/s00425-015-2389-y (PMC4698290; doi:10.1007/s00425-015-2389-y)
Supplement: Supplementary file 10 — Supplementary material 10 (DOCX 17 kb) [file 425_2015_2389_MOESM10_ESM.docx]

**Table S5** The distribution of pi value, ka, ks for *Cercis gigantea* miRNA targets

| **Target mRNA** | **Length** | **Length(reading frame)** | **Mapped Length** | ***G. max* CDS ID** | **Length** | **Pi value** | **Ks** | **Ka** | **Ka/Ks** |
| --- | --- | --- | --- | --- | --- | --- | --- | --- | --- |
| Conserved target |  |  |  |  |  |  |  |  |  |
| gi\|691493380 | 1668 | 1026 | 1026 | GLYMA12G36540.7 | 912 | 0.24 | 0.452 | 0.209 | 0.462 |
| gi\|691492924 | 1744 | 1131 | 1131 | GLYMA02G05450.1 | 1113 | 0.159 | 1.56 | 0.108 | 0.069 |
| gi\|691492802 | 828 | 372 | 372 | GLYMA08G13510.1 | 366 | 0.125 | 0.624 | 0.136 | 0.217 |
| gi\|691490548 | 941 | 342 | 342 | GLYMA12G16390.1 | 342 | 0.045 | 0.487 | 0.033 | 0.068 |
| gi\|691488591 | 648 | 267 | 267 | GLYMA09G17255.1 | 270 | 0.236 | 0.485 | 0.143 | 0.295 |
| gi\|691490413 | 1722 | 1134 | 1134 | GLYMA12G35701.1 | 1242 | 0.213 | 0.506 | 0.207 | 0.409 |
| gi\|691487022 | 1901 | 600 | 576 | GLYMA06G19680.1 | 579 | 0.065 | 0.485 | 0.053 | 0.109 |
| gi\|691485626 | 1901 | 963 | 948 | GLYMA09G34565.1 | 1014 | 0.225 | 0.722 | 0.199 | 0.275 |
| gi\|691480627 | 1416 | 861 | 861 | GLYMA19G02370.5 | 861 | 0.043 | 0.651 | 0.032 | 0.049 |
| gi\|691480372 | 2184 | 1824 | 1788 | GLYMA07G29000.1 | 1806 | 0.225 | 0.773 | 0.207 | 0.268 |
| gi\|691479452 | 1886 | 834 | 816 | GLYMA02G09600.1 | 1251 | 0.257 | 0.878 | 0.228 | 0.26 |
| gi\|691479675 | 2465 | 1965 | 1476 | GLYMA13G02080.2 | 1476 | 0.146 | 0.54 | 0.099 | 0.18 |
| gi\|691477365 | 2507 | 1644 | 1569 | GLYMA13G25716.2 | 1611 | 0.154 | 0.44 | 0.139 | 0.316 |
| gi\|691475213 | 2657 | 1755 | 1755 | GLYMA19G39420.1 | 1764 | 0.083 | 0.571 | 0.072 | 0.126 |
| gi\|691474734 | 979 | 537 | 537 | GLYMA13G40090.1 | 465 | 0.225 | 1.183 | 0.199 | 0.168 |
| gi\|691466148 | 2446 | 1257 | 1128 | GLYMA04G43350.1 | 1689 | 0.251 | 0.671 | 0.226 | 0.337 |
| gi\|691465468 | 2285 | 1809 | 1809 | GLYMA10G35481.1 | 1839 | 0.148 | 0.706 | 0.124 | 0.175 |
| gi\|691465470 | 3261 | 2121 | 2121 | GLYMA12G29720.1 | 2103 | 0.166 | 1.192 | 0.111 | 0.093 |
| gi\|691465196 | 1419 | 1014 | 1014 | GLYMA01G33270.1 | 2241 | 0.123 | 0.909 | 0.115 | 0.126 |
| gi\|691463335 | 3218 | 1923 | 1923 | GLYMA08G07970.1 | 1920 | 0.106 | 0.471 | 0.096 | 0.204 |
| gi\|691462079 | 2088 | 1152 | 1152 | GLYMA19G26390.1 | 1098 | 0.202 | 0.869 | 0.182 | 0.209 |
| gi\|691461032 | 2171 | 1371 | 1050 | GLYMA01G34650.2 | 1377 | 0.266 | 0.524 | 0.242 | 0.462 |
| gi\|691460139 | 1078 | 765 | 765 | GLYMA13G05540.1 | 870 | 0.159 | 0.618 | 0.172 | 0.278 |
| gi\|691459251 | 3960 | 2694 | 2694 | GLYMA15G09750.3 | 2697 | 0.069 | 0.49 | 0.052 | 0.106 |
| gi\|691459148 | 3063 | 2217 | 2217 | GLYMA01G18040.1 | 2235 | 0.175 | 0.765 | 0.139 | 0.182 |
| gi\|691457013 | 2011 | 1191 | 1191 | GLYMA18G00870.1 | 1191 | 0.071 | 0.472 | 0.057 | 0.121 |
| gi\|691455724 | 1890 | 1689 | 1689 | GLYMA09G12030.1 | 1671 | 0.197 | 0.774 | 0.151 | 0.195 |
| gi\|691451897 | 3586 | 1320 | 1320 | GLYMA12G20160.2 | 1194 | 0.368 | 1.357 | 0.31 | 0.228 |
| gi\|691452741 | 2019 | 1431 | 1431 | GLYMA12G35990.1 | 1431 | 0.03 | 0.467 | 0.007 | 0.015 |
| gi\|691451139 | 3810 | 2826 | 2826 | GLYMA01G44491.2 | 2859 | 0.273 | 0.448 | 0.252 | 0.562 |
| gi\|691450023 | 1157 | 714 | 621 | GLYMA08G18640.1 | 660 | 0.188 | 0.843 | 0.129 | 0.153 |
| gi\|691449279 | 3596 | 3177 | 3177 | GLYMA09G29720.1 | 3177 | 0.082 | 0.883 | 0.049 | 0.055 |
| gi\|691448577 | 2730 | 1683 | 1683 | GLYMA04G32002.1 | 1695 | 0.138 | 0.666 | 0.124 | 0.186 |
| gi\|691447416 | 4733 | 2175 | 2175 | GLYMA16G23150.3 | 2121 | 0.25 | 0.631 | 0.213 | 0.337 |
| gi\|691443742 | 1216 | 819 | 819 | GLYMA18G40560.1 | 801 | 0.307 | 0.642 | 0.242 | 0.377 |
| gi\|691466862 | 2869 | 2085 | 2085 | GLYMA16G29440.4 | 2088 | 0.115 | 0.501 | 0.068 | 0.136 |
| gi\|691442095 | 4622 | 2541 | 2541 | GLYMA09G02750.1 | 2559 | 0.075 | 0.523 | 0.04 | 0.076 |
| gi\|691442087 | 2541 | 2268 | 2268 | GLYMA11G20520.1 | 2529 | 0.074 | 0.524 | 0.037 | 0.071 |
| gi\|691435294 | 3644 | 3222 | 3141 | GLYMA03G14615.1 | 3384 | 0.378 | 0.866 | 0.312 | 0.36 |
| gi\|691435270 | 1967 | 1743 | 1743 | GLYMA06G46830.1 | 1743 | 0.33 | 0.786 | 0.264 | 0.336 |
| Nonconserved  target |  |  |  |  |  |  |  |  |  |
| gi\|691496673 | 1648 | 1239 | 1239 | GLYMA07G35561.1 | 1269 | 0.32 | 1.848 | 0.365 | 0.198 |
| gi\|691484799 | 1057 | 786 | 786 | GLYMA16G00920.1 | 810 | 0.196 | 0.674 | 0.17 | 0.252 |
| gi\|691484368 | 1221 | 960 | 954 | GLYMA17G05831.1 | 924 | 0.217 | 0.899 | 0.17 | 0.189 |
| gi\|691478191 | 1200 | 852 | 849 | GLYMA13G16890.1 | 915 | 0.554 | 2.478 | 0.491 | 0.198 |
| gi\|691470779 | 2064 | 1500 | 1500 | GLYMA01G45290.1 | 1143 | 0.159 | 1.345 | 0.11 | 0.082 |
| gi\|691469139 | 1334 | 585 | 585 | GLYMA05G04900.1 | 606 | 0.164 | 0.737 | 0.155 | 0.21 |
| gi\|691466946 | 1551 | 1227 | 1227 | GLYMA08G16190.1 | 1353 | 0.213 | 0.631 | 0.175 | 0.277 |
| gi\|691455866 | 2355 | 1665 | 1665 | GLYMA19G01630.2 | 1707 | 0.298 | 1.252 | 0.229 | 0.183 |
| gi\|691465333 | 2744 | 1794 | 1794 | GLYMA18G44960.1 | 1797 | 0.086 | 0.532 | 0.055 | 0.103 |
| gi\|691475785 | 1499 | 1197 | 1197 | GLYMA08G39920.1 | 1203 | 0.302 | 0.661 | 0.292 | 0.442 |
